# Dominant TET2 mutations predict adverse prognosis in cytogenetically normal acute myeloid leukemia patients

**DOI:** 10.3389/fonc.2025.1730830

**Published:** 2026-01-05

**Authors:** Zhuanghui Hao, Jingjing Xia, Sicheng Bian, Miaoke Song, Miao Zhang, Jingyi Feng, Shuo Li, Huichao Wang, Yaofang Zhang, Wanfang Yang, Jianmei Chang, Fanggang Ren, Xufeng Huang, Xiuhua Chen, Hongwei Wang

**Affiliations:** 1 Institute of Hematology, The Second Hospital of Shanxi Medical University, Taiyuan, China; 2 Department of Medical Genetics, Shanxi Medical University, Taiyuan, Shanxi, China; 3 Department of Medicine, MetroHealth System, Case Western Reserve University, Cleveland, OH, United States; 4 Department of Basic Medicine, Shanxi University of Chinese Medicine, Jinzhong, China; 5 Department of Data Visualization, Faculty of Informatics, University of Debrecen, Debrecen, Hungary

**Keywords:** acute myeloid leukemia, co-mutations, dominant, outcome, *TET2* mutation, variant allele frequency

## Abstract

**Objective:**

This study aimed to characterize TET2 mutations in CN-AML, assess their clinical features, and evaluate the prognostic impact of VAF and clonal hierarchy on overall survival (OS) and relapse-free survival (RFS).

**Methods:**

A cohort of 206 adult CN-AML patients was analyzed for the presence of *TET2* mutation characteristics, variant allele frequency (VAF) and clonal status. Clinical and prognostic implications were evaluated through survival analyses and validated by the Beat AML public database.

**Results:**

*TET2* mutations were detected in 18.9% of CN-AML patients, with a median age of 55 years, significantly older than *TET2* wild-type patients (*P* < 0.001). OS and RFS were no difference in the high-VAF group and low-VAF group. Patients with dominant *TET2* mutations exhibited significantly shorter OS and RFS compared to subclonal group (*P* < 0.05). Multivariate Cox regression identified dominant *TET2* mutations as an independent adverse prognostic factor for OS (HR = 2.026,*P* = 0.039). A nomogram model based on these findings demonstrated robust predictive performance (AUC = 0.735) and was validated by the Beat AML database.

**Conclusions:**

The prognostic impact of *TET2* mutations is not determined by VAF, but rather by *TET2* clonal dominance and the interplay between mutations within the same clone.

## 1 Introduction

Acute myeloid leukemia (AML) is a heterogeneous hematologic malignancy characterized by diverse genetic and epigenetic alterations that influence disease progression and prognosis (1–3). Among patients with cytogenetically normal AML (CN-AML), which constitutes approximately 50% of AML cases, gene mutations play a crucial role in risk stratification and therapeutic decision-making (4, 5). Therefore, identifying molecular markers closely associated with the prognosis of CN-AML is of critical importance for optimizing treatment strategies and prognostic evaluation (6, 7).

Among the various genetic alterations implicated in AML, ten-eleven translocation 2 (*TET2*) mutations have garnered attention due to its high prevalence in myeloid malignancies and its impact on DNA demethylation and epigenetic regulation (8, 9). *TET2* mutations are detected in approximately 10–25% of AML patients and are frequently observed in older individuals (10). However, their prognostic significance in CN-AML remains controversial. While some studies have suggested an association between *TET2* mutations and adverse outcomes, the evidence remains inconclusive (11–14) Furthermore, the clonal status of *TET2* mutations—whether dominant or subclonal—and their mutation burden, as reflected by variant allele frequency (VAF), have not been systematically studied in CN-AML.

In this study, we aimed to comprehensively evaluate the clinical and prognostic relevance of *TET2* mutations in a well-defined cohort of 206 adult CN-AML patients. We then investigated the impact of *TET2* clonal status and mutation burden on clinical outcomes. Understanding these aspects is critical for elucidating the clinical implications of *TET2* mutations in CN-AML.

## 2 Materials and methods

### 2.1 Patients and samples

We collected clinical data and bone marrow samples from 206 newly diagnosed cytogenetically normal acute myeloid leukemia (CN-AML) patients treated at the Second Hospital of Shanxi Medical University between February 2017 and December 2020. Data included age, sex, hematologic parameters, bone marrow blast percentage, and history of prior chemotherapy or radiotherapy, all derived from medical records at diagnosis. Patients were treated according to the 2017 European LeukemiaNet (ELN) recommendations for diagnosis and management of AML. Of the 206 patients, 145 received high-intensity induction chemotherapy (the “3 + 7” regimen), consisting of cytarabine (Ara-C) combined with daunorubicin or idarubicin, aiming for complete remission (CR) (15). The remaining 61 patients, primarily elderly or those with comorbidities, received a lower-intensity induction regimen (GAG protocol) to mitigate treatment-related toxicities. Treatment doses and cycles were tailored to individual patient needs, with regular monitoring and adjustments during therapy to ensure optimal outcomes.

Clinical endpoints were defined following the 2017 ELN recommendations (15). First complete remission (CR1) was defined as the absence of leukemia cells in peripheral blood and ≤5% blasts in bone marrow (excluding other causes such as post-consolidation bone marrow regeneration) or the absence of extramedullary leukemia. Relapse (RP1) was defined as the reappearance of leukemic blasts in peripheral blood, ≥5% blasts in the bone marrow, or extramedullary involvement after achieving CR1. Overall survival (OS) was defined as the time from diagnosis to death or last follow-up, whereas relapse-free survival (RFS) was measured from the date of CR1 to relapse, death, or last follow-up. In addition, five patients with *TET2* mutations underwent longitudinal monitoring using NGS at multiple disease stages, including initial diagnosis, remission, and relapse. This approach allowed for a comprehensive assessment of clonal evolution and molecular dynamics over the course of the disease.

All samples were collected with informed consent from patients. The study adhered to the principles of the Declaration of Helsinki and was approved by the Ethics Committee of the Second Hospital of Shanxi Medical University ((2024)YX NO.362).

### 2.2 Molecular analysis

Targeted next-generation sequencing (NGS) was performed on fresh bone marrow samples obtained at the time of diagnosis. Genomic DNA was extracted via the Mini Blood DNA Kit (Qiagen or OMEGA) and quantified with a NanoDrop spectrophotometer. We analyzed mutational hotspots or entire coding regions of 34 genes associated with myeloid malignancies, including *FLT3*, *JAK2*, *KIT*, *MPL*, *CALR*, *CSF3R*, *PDGFRA*, *CEBPA*, *NRAS*, *KRAS*, *NPM1*, *TP53*, *RUNX1*, *GATA2*, *WT1*, *TET2*, *DNMT3A*, *IDH1*, *IDH2*, *ASXL1*, *BCOR*, *BCORL*, *CBL*, *ETV6*, *EZH2*, *MLL*, *NOTCH2*, *PHF6*, *SF3B1*, *SRSF2*, *SH2B3*, *SETBP1*, *U2AF1*, and *ZRSR2*. Briefly, 50 ng of genomic DNA was used for each reaction. The samples were sequenced on the MiSeq platform (Illumina), with an average sequencing depth of 2000×. The variant allele frequency (VAF) was calculated as the percentage of reads with a specific DNA variant compared to the total coverage at the site. Mutations were considered present if the VAF exceeded 5% (5, 16). Patients harboring two distinct pathogenic TET2 variants in the same sample were classified as having double distinct TET2 mutation sites.

### 2.3 Dominant and subclonal analysis of *TET2* Mutations

To distinguish between dominant and subclonal *TET2* mutations, we adopted a clonal analysis strategy previously established for *PTPN11* mutations (17). Specifically, we compared the VAF of *TET2* mutations with those of co-mutated genes to infer their clonal hierarchy. For a mutation to be classified as subclonal, a minimum difference of ≥10% VAF compared to the most prominent co-mutation was required. Conversely, *TET2* mutations with VAFs higher than or similar to (<10% difference) co-mutations were classified as dominant (18, 19). Sensitivity analyses using 5% or 15% cutoffs did not materially change subgroup assignment in this cohort. Beat Acute Myeloid Leukemia (Beat AML) was accessed on Data from https://registry.opendata.aws/beataml. 

### 2.4 Data source and patient selection

Data for this study were obtained from the Beat Acute Myeloid Leukemia (Beat AML) dataset, accessed via the Registry of Open Data on AWS (https://registry.opendata.aws/beataml). A total of 256 patients with normal karyotype acute myeloid leukemia AML were selected from the dataset. Clonal hierarchy stratification was performed based on VAF values of patient-specific mutations, following the classification criteria described above.

### 2.5 Prediction of mutational pathogenicity

The pathogenicity of missense variants was assessed using multiple prediction tools, including MutationTaster (https://www.mutationtaster.org/), AlphaMissense (https://alphamissense.hegelab.org/), PolyPhen-2 (http://genetics.bwh.Harvard.edu/pph2/), and SIFT (https://sift.bii.a-star.edu.sg/) (20–22).

### 2.6 Statistical analysis

Categorical variables were summarized using frequencies and percentages, while continuous variables were presented as medians with ranges. Group comparisons for categorical variables were performed using the Chi-square test or Fisher’s exact test, and continuous variables were compared using the Mann-Whitney U test. A two-sided p-value <0.05 was considered statistically significant. Kaplan-Meier survival curves for OS and RFS were generated, and differences between groups were assessed using the log-rank test. Multivariate analysis of prognostic factors was conducted using a Cox proportional hazards model.

All statistical analyses were performed using SPSS software version 25.0. Graphical presentations were created with GraphPad Prism™ 8.30, and nomograms with internal validation were constructed using the R package stats version4.4.1.

## 3 Results

### 3.1 Clinical characteristics of patients with TET2-mutated CN-AML

**Figure 1:**
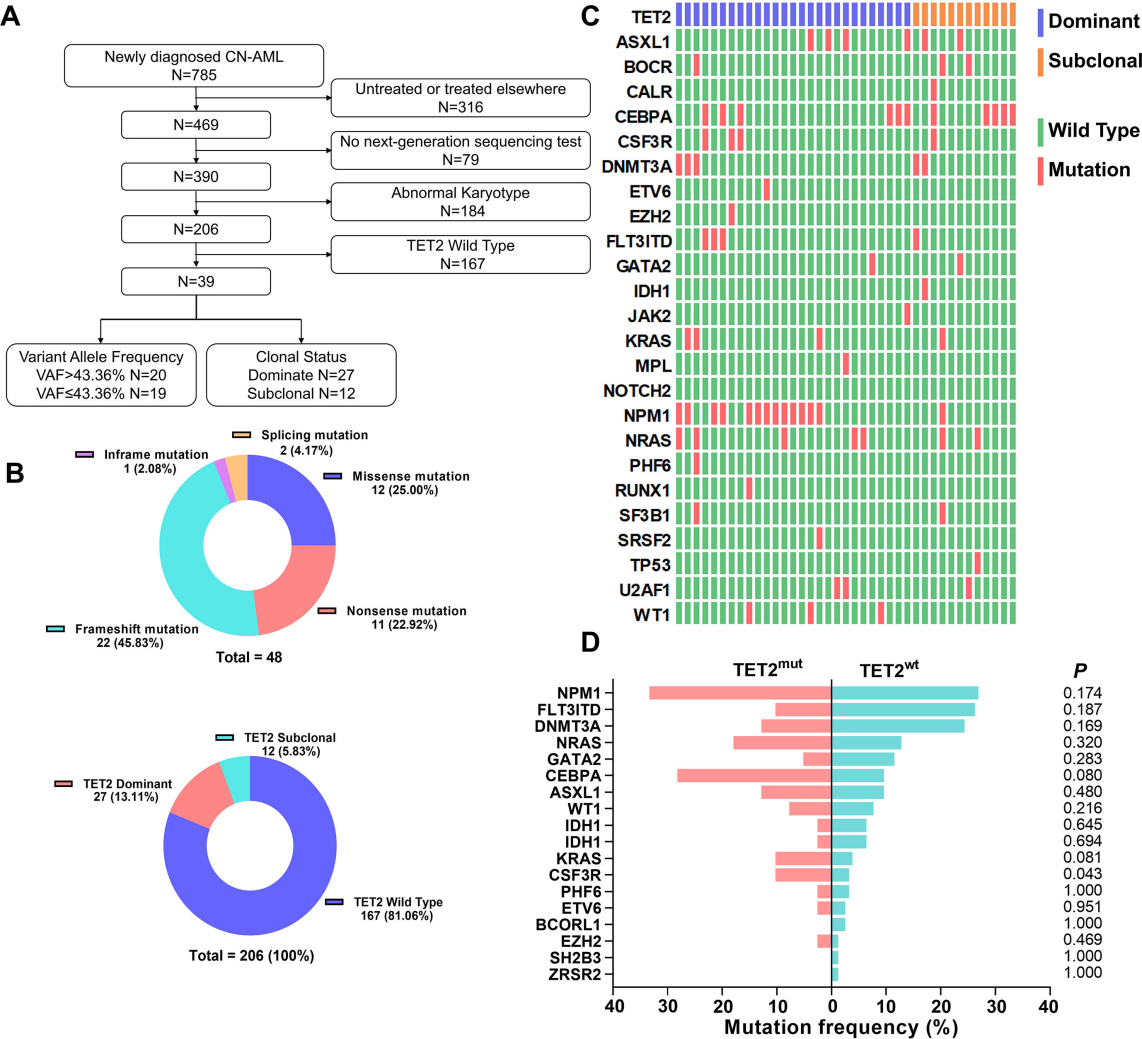
*TET2* mutation types, co-mutation profiles, and clonal subpopulation proportions in 39 patients. **(A)** The process of patient recruitment and cohort assignment in this study. **(B)** Distribution of *TET2* mutational site and clonal status in 39 *TET2*-mutated patients. **(C)** Mutation heatmap at *TET2* mutation patients **(D)** and comparison of co-mutation frequencies between *TET2*-mutated and wild-type groups.

A total of 785 patients with newly diagnosed AML were enrolled from February 2017 to December 2020 at our institute. Our study enrolled participants who met the following eligibility criteria: (1) received induction or consolidation chemotherapy at our hospital; (2) had comprehensive examination and case data, including NGS data; and (3) had a normal karyotype (**Figure 1A**). We ultimately selected 206 patients, among whom 39 (18.9%) carried *TET2* mutations. The median age of *TET2*-mutated patients was 55 years (range: 19–78 years), significantly older than the median age of *TET2* wild-type patients (47 years, range: 12–86 years; P < 0.001), consistent with previous findings. There were no significant differences between *TET2*-mutated and *TET2* wild-type patients in terms of red blood cell count, white blood cell count, hemoglobin level, platelet count, bone marrow blast percentage, lactate dehydrogenase (LDH), or European LeukemiaNet (ELN) risk stratification (P > 0.05 for all comparisons, **Table 1**). Detailed clinical characteristics are presented in **Table 1**. Mutation site analysis in the 39 patients with TET2 mutations revealed 30 (76.9%) cases with single mutations and 9 (23.1%) carried double distinct TET2 mutations sites. The mutations were predominantly located in exons 3–11, which are recognized as TET2 mutation hotspots. The mutation types included missense (12/48), frameshift (22/48), nonsense (11/48), insertions (1/48), and splice-site mutations (2/48) (**Figure 1B**). Pathogenicity prediction using tools such as MutationTaster, AlphaMissense, PolyPhen-2, and SIFT revealed that all TET2 mutations, except for the F775L variant, were classified as pathogenic (**Supplementary Table S1**).The median VAF of TET2 mutations was 43.36% (range: 5.58%–96.58%). Through VAF analysis for each patient, the clonal status of TET2 mutations was inferred, with 27 cases classified as dominant and 12 as subclonal (**Figure 1B**).

**Table 1:** The clinical characteristic of *TET2* mutation in CN-AML.

Characteristics	TET2mut N=39	TET2wt N=167	*P*
Age, *n* (%)			**<0.001**
<60	16 (41.1)	123 (73.6)	
≥60	23 (58.9)	44 (26.4)	
Sex, *n* (%)			**0.492**
male	22 (56.5)	84(49.7)	
female	17 (43.5)	83(50.3)	
Laboratory data, median (range)			
RBC (×10^12^/L)	2.49 (1.17-4.37)	2.37 (0.92-4.94)	**0.919**
Hb (g/L)	77 (46-136)	80 (24-157.2)	**0.457**
WBC (×10^9^/L)	11.8 (0.89-207.48)	11.23 (0.42-283.79)	**0.902**
PLT (×10^9^/L)	29 (5-498)	43 (-4-317)	**0.344**
BM blast (%)	55 (20-93)	57 (20-97)	**0.349**
LDH (U/mL)	370 (95-2544)	396 (140-3935)	**0.992**
2022 ELN risk classification, *n* (%)			**0.600**
Favorable	3 (7.7)	19 (11.4)	
Intermediate	28 (71.8)	123 (73.7)	
Adverse	8 (20.5)	25 (14.9)	
Outcome			
CR1, *n* (%)	26 (66.7)	131 (84.0)	**0.104**
RP1, *n* (%)	17 (65.4)	46 (35.1)	**0.004**
OS, median (range)	24 (2-47)	40 (1-60)	**0.017**
RFS, median (range)	14 (6-35)	17 (1-56)	**0.036**

RBC, Red blood cell, WBC, white blood cell, PLT, platelet, LDH, Lactate dehydrogenase, HBDH, -Hydroxybutyrate Dehydrogenase, ELN, European Leukemia Network, CR1, First Complete Remission, RP1,First Recurrence, OS, Overall Survival, RFS:Relapse-Free Survival. Bold values indicate p-values with statistical significance (P < 0.05).

Additionally, concurrent mutations in patients with TET2-mutations included NPM1 (33.3%), CEBPA (28.2%), NRAS (17.9%), ASXL1 (15.3%), DNMT3A (12.8%), FLT3 (10.2%), KRAS (10.2%), and CSF3R (10.2%). Compared with TET2 wild-type patients, patients with TET2-mutations exhibited a significantly higher frequency of CSF3R mutations (10.2% vs. 3.0%, P = 0.043) (**Figures 1C,D**).

### 3.2 Prognostic characteristics of TET2 and co-mutations in CN-AML

**Figure 2:**
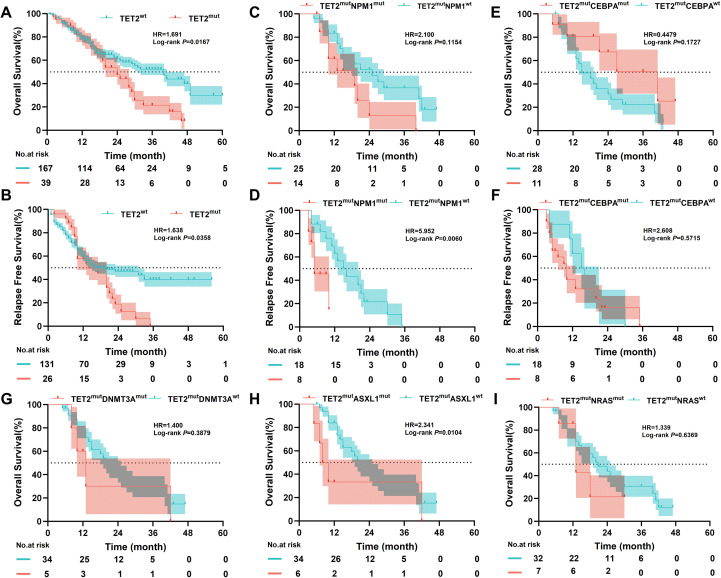
Prognostic impact of TET2 mutations and co-mutations in CN-AML. **(A, B)** Kaplan–Meier curves for overall survival (OS) and relapse-free survival (RFS) in TET2-mutated (n=39) versus TET2 wild-type (n=167) patients. TET2 mutations were associated with inferior OS and RFS. **(C–I)** Kaplan–Meier curves for OS and/or RFS in TET2-mutated patients stratified by co-mutational status: NPM1 **(C, D)**, CEBPA **(E, F)**, DNMT3A **(G)**, ASXL1 **(H)**, and NRAS **(I)**.

Prognostic analysis of all 206 patients revealed that those carrying TET2 mutations had significantly shorter OS and RFS than those with wild-type TET2 (**Figures 2A,B**). We further analyzed the factors influencing the prognosis of patients with TET2 mutations. Since all patients with TET2 mutations were accompanied by concomitant mutations, we first considered the impact of these co-mutations. Given the relatively high mutation frequencies of NPM1, CEBPA, NRAS, ASXL1, and DNMT3A, we further evaluated their effects on prognosis. The results indicated that TET2/NPM1 co-mutations had no significant impact on OS in patients with TET2 mutations but significantly shortened RFS (OS: 19 vs. 25 months, P = 0.115; RFS: 5 vs. 16 months, P = 0.006) (**Figures 2C,D**). In contrast, TET2/CEBPA co-mutations had no significant impact on either OS or RFS (OS: 41 vs. 15 months, P = 0.173; RFS: 10 vs. 15.5 months, P = 0.572) (**Figures 2E,F**). We further analyzed the prognostic impact of TET2 co-mutations with NRAS, ASXL1, and DNMT3A separately. The results showed that patients with TET2^mu^tASXL1^mut^ had significantly shorter OS compared with the TET2^mut^ASXL1^wt^ group (9 vs. 21 months, P = 0.010), whereas patients with TET2^mut^DNMT3A^mut^ showed no difference in OS compared with the TET2^mut^DNMT3A^wt^ group (13 vs. 21 months, P = 0.388), and patients with TET2^mut^NRAS^mut^ also exhibited no significant difference in OS compared with the TET2^mut^NRAS^wt^ group (13 vs. 21 months, P = 0.670) (**Figures 2G–I**).

### 3.3 Clinical impact of TET2 mutation number and burden in CN-AML

**Figure 3:**
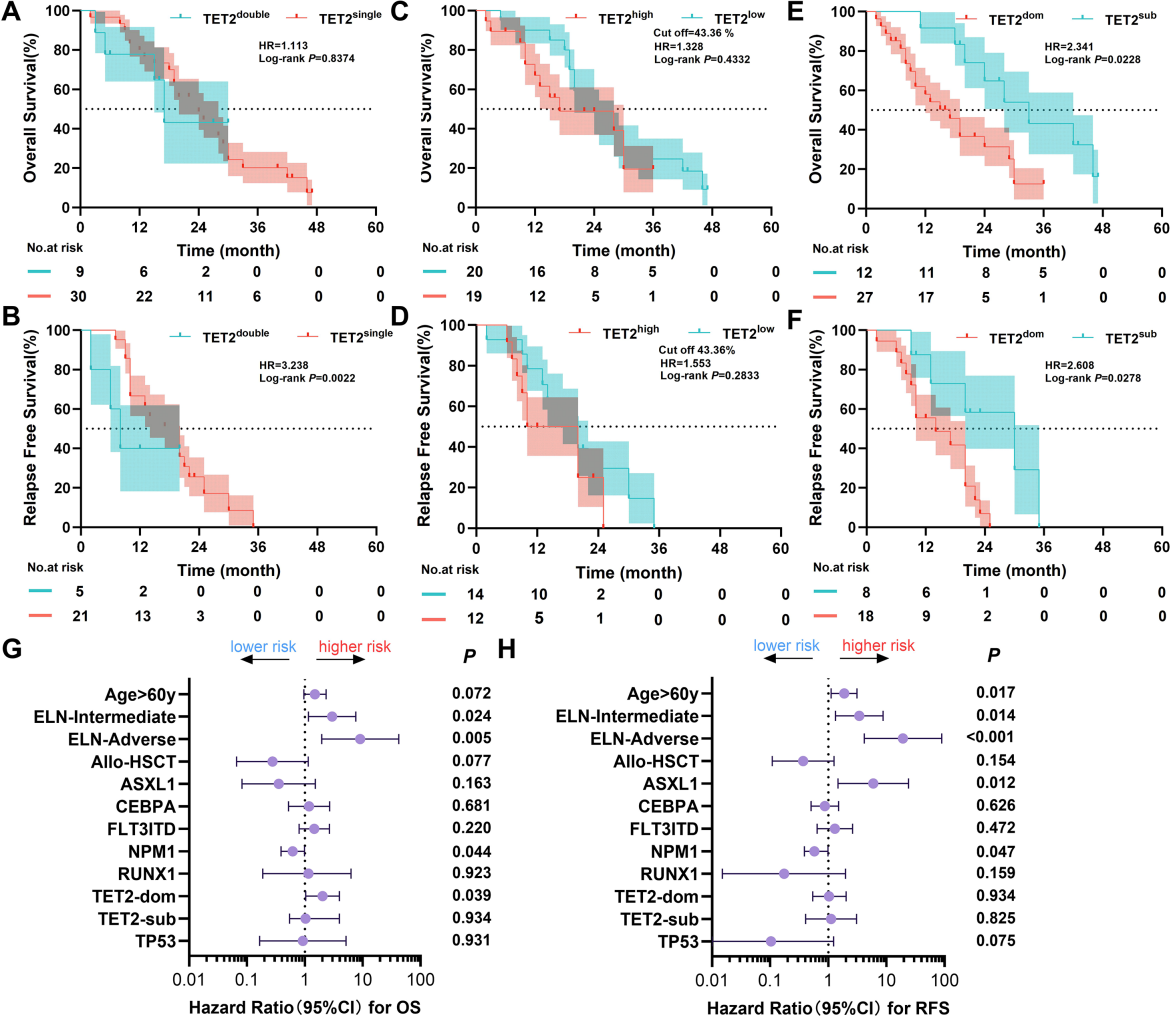
Impact of TET2 mutation multiplicitym, different VAF and mutational status on prognosis in CN-AML. **(A, B)** Kaplan–Meier curves for OS and RFS in patients with single TET2 mutation (n=30) versus double distinct TET2 mutation sites(n=9). Double mutation sites group were associated with inferior RFS but not OS. **(C, D)** Kaplan–Meier curves for OS and RFS stratified by TET2 VAF (>43.36%, n=20; ≤43.36%, n=19). No significant differences were observed. **(E, F)** Kaplan–Meier curves for OS and RFS in patients with dominant (n=27) versus subclonal (n=11) TET2 mutations. Dominant mutations were associated with inferior OS and RFS. **(G, H)** Multivariate Cox regression forest plots for OS **(G)** and RFS **(H)**, adjusted for age >60 years, ELN risk, allo-HSCT, and mutations in NPM1, ASXL1, CEBPA, FLT3-ITD, RUNX1, and TP53. Dominant TET2 mutations predicted inferior OS but not RFS.

Patients were stratified by mutation multiplicity into single-mutation (n=30) and double distinct mutation sites(n=9) groups. Baseline clinical features, including age, white blood cell count, hemoglobin level, platelet count, bone marrow blast percentage, lactate dehydrogenase level, and ELN risk category, did not differ significantly between groups (P>0.05 for all). Patients with double distinct TET2 mutations sites(n=9) exhibited inferior relapse-free survival compared with single-mutation cases (median RFS 13 vs 21 months; P = 0.022), but no significant difference in overall survival (**Figures 3A,B**).

Based on the median VAF 43.36% of TET2 mutations, with the higher VAF used for patients with dual mutations, the 39 patients with TET2 mutation were further divided into high-VAF (>43.36%, n = 20) and low-VAF (≤43.36%, n = 19) groups. The high-VAF group exhibited significantly higher white blood cell counts than the low-VAF group (23.01 vs. 6.10 ×109/L, P = 0.013). No significant differences were observed between the two groups in other parameters, including red blood cell count, hemoglobin level, platelet count, bone marrow blast percentage, LDH, or ELN risk stratification (**Supplementary Table S2**). Further analysis revealed that OS and RFS were no difference in the high-VAF group and the low-VAF group (**Figures 3C,D**).

### 3.4 Clinical impact of TET2 clonal status in CN-AML

**Figure 4:**
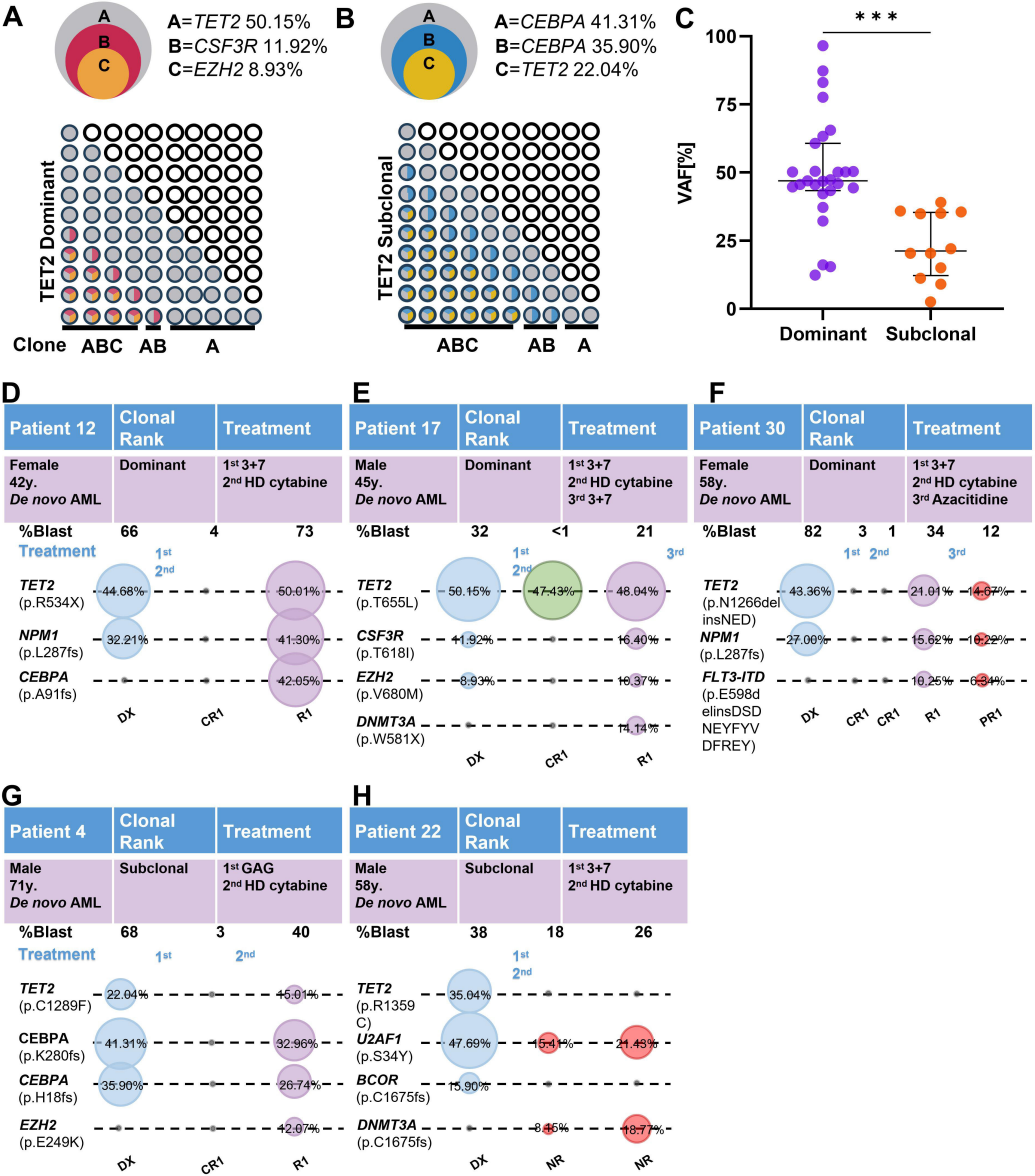
Clonal evolution in TET2-mutated patients. **(A, B)** Schematic illustration of representative clonal hierarchies (depicted from VAFs of TET2 mutations and comutations) in TET2dom **(A)** and TET2sub **(B)** AMLs of 2 TET2-mutated patients (overlapping circles, top) and potential sequential acquisition of TET2 mutations (and co-mutations) as early (TET2dom) or late (TET2sub) event during clonal evolution (grid of circles, bottom). **(C)** VAFs of dominant (n = 27) and subclonal (n = 12) TET2 mutations. **(D–F)** Linear evolution patterns were observed in three patients with dominant TET2 clones. **(G–H)** Among the two subclonal patients, one exhibited linear evolution, while the other showed branching evolution.

We classified TET2 mutations into dominant (TET2dom, n = 27) and subclonal (TET2sub, n = 12) categories on the basis of VAF comparisons with comutated genes. A schematic representation of typical clonal configurations for TET2 dominant clones and TET2 subclones AMLs is shown in **Figures 4A, B**. A comparison of clinical characteristics between TET2dom and TET2sub revealed that the dominant clone group had significantly higher white blood cell counts than the subclone group (36.63 vs. 10.03, P = 0.026). No significant differences were observed between the two groups in other laboratory parameters such as the red blood cell count, hemoglobin level, platelet count, or bone marrow blast percentage (**Table 2**). Comparative VAF analysis demonstrated significantly higher TET2 VAFs in the TET2dom cohort compared with the TET2sub cohort (**Figure 4C**).

**Table 2:** The clinical characteristic of *TET2* clonal rank in CN-AML.

Characteristics	TET2dom N=27	TET2sub N=12	*P*
Age, *n* (%)			**0.219**
<60	13 (48.1)	3 (25.0)	
≥60	14 (51.9)	9 (75.0)	
Sex, *n* (%)			**0.119**
male	13 (48.1)	9 (75.0)	
female	14 (51.9)	3 (25.0)	
Laboratory data, median (range)			
RBC (×10^12^/L)	2.58 (1.24-4.37)	2.42 (1.17-3.86)	**0.856**
Hb (g/L)	77 (46-136)	79 (46-123)	**0.394**
WBC (×10^9^/L)	6.10 (0.89-115.20)	23.01 (2.81-207.48)	**0.026**
PLT (×10^9^/L)	28 (6-269)	43 (5-297)	**0.262**
BM blast (%)	55 (21-93)	54 (20-85)	**0.496**
LDH (U/mL)	359 (95-1920)	401 (203-2544)	**0.448**
2022 ELN risk classification, *n* (%)			**0.664**
Favorable	2 (10.5)	1 (5.0)	
Intermediate	14 (73.7)	14 (70.0)	
Adverse	3 (15.8)	5 (25.0)	
Outcome			
CR1, *n* (%)	18 (66.7)	8 (66.7)	**1.000**
RP1, *n* (%)	11 (61.1)	6 (75.0)	**0.667**
OS, median (range)	19 (2-36)	33 (15-47)	**0.023**
RFS, median (range)	14 (2-25)	30 (9-35)	**0.028**

Dom, dominate clonal; sub, subclonal; RBC, Red blood cell; WBC, white blood cell; PLT, platelet; LDH, Lactate dehydrogenase; HBDH, Hydroxybutyrate Dehydrogenase; ELN, European Leukemia Network; CR1, First Complete Remission; RP1, First Recurrence; OS, Overall Survival; RFS, Relapse-Free survival.

Bold values indicate p-values with statistical significance (P < 0.05).

Longitudinal NGS was performed in five TET2-mutated patients at diagnosis, remission, and relapse (**Figures 4D–H**). Among the three patients with dominant TET2 mutations, linear evolution with acquisition of additional mutations at relapse was observed. Among the two patients with subclonal TET2 mutations, one showed linear and one showed branched evolution. Because of the extremely small number of patients (n=5), no conclusions can be drawn regarding putative differences in evolutionary patterns between dominant and subclonal TET2 mutations. These observations are presented solely for illustrative purposes (23–26).

Further survival analysis demonstrated that patients with dominant TET2 mutations had significantly shorter OS (P = 0.023) and RFS (P = 0.028) than those with subclonal mutations (**Figures 3E,F**). Univariate Cox regression analyses identified variables with P < 0.1, along with known prognostic factors (e.g., age > 60 years, ELN risk classification, transplantation, TET2 clonal status, and ASXL1, CEBPA, FLT3-ITD, NPM1, RUNX1, and TP53 mutations), which were included in a multivariate Cox regression model. The analysis identified dominant TET2 mutations as an independent adverse prognostic factor for OS (HR [95% CI] = 2.026 [1.036–3.964], P = 0.039); however, it was not an independent factor for RFS (HR [95% CI] = 1.120 [0.409–3.066], P = 0.825) (**Supplementary Tables S3**, **Supplementary Tables S4**); **Figures 3G,H**).

### 3.5 Nomogram model and external validation

**Figure 5:**
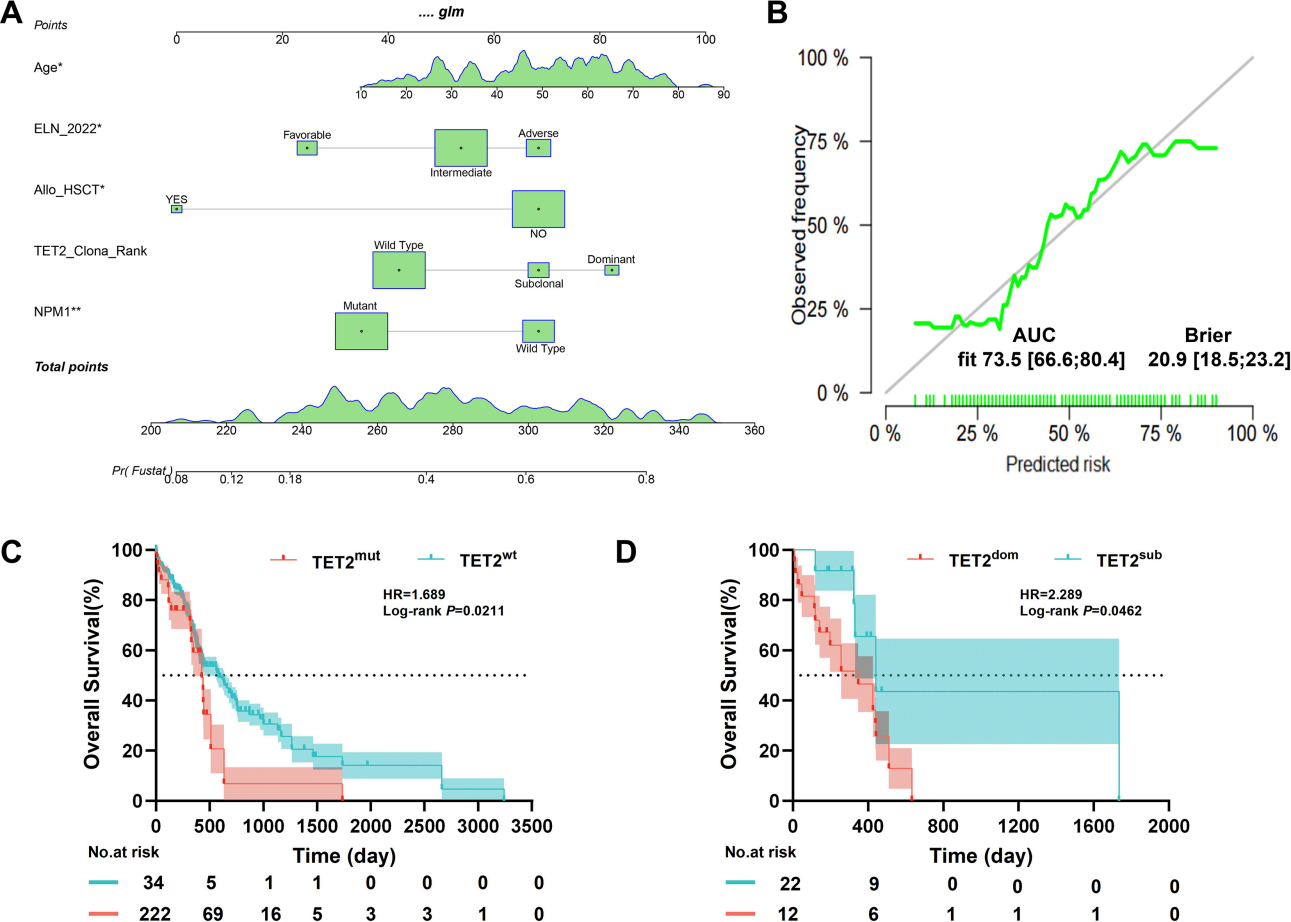
Nomogram and evaluation of predictive model performance. **(A)** Nomogram for predicting prognosis in all AML patients within the study cohort. The vertical line extending to the “Points” bar helps calculate individual patient scores. The total score is used to predict 5 year OS from the “Total Points” line. **(B)** Calibration curve for predicting survival probability in the study cohort. The gray line represents the ideal survival probability, and the green line shows the actual calibration results. The model^rsquo;s AUC of 0.735 indicates high accuracy in predicting survival probabilities for AML patients. **(C, D)** Validation of TET2 mutation’s prognostic impact in 256 CN-AML patients using the Beat AML database..

Based on the results of univariate and multivariate Cox regression analyses, we constructed a nomogram model to predict survival probabilities in patients with AML. The model integrates age, ELN risk stratification, allo-HSCT, NPM1 mutation, and TET2 clonal status. Internal validation was performed using random sampling (**Figure 5A**). A nomogram based on the Cox proportional hazards model was developed to facilitate individualized prediction of 5-year OS in patients with AML. The model demonstrated a concordance index (C-index) of 0.735 (95% CI: 0.666–0.804), indicating good predictive accuracy. Calibration curves a high degree of agreement between the predicted and actual survival probabilities, confirming the model’s reliability (**Figure 5B**). This nomogram serves as a convenient and practical tool for personalized prognosis assessment in clinical settings.

To validate the findings, we utilized data from the Beat AML public database to independently assess the impact of TET2 mutations on OS in patients with CN-AML. The validation analysis confirmed that patients with TET2 mutations had significantly shorter OS than those with wild-type TET2 (**Figure 5C**). Subgroup analysis based on clonal status further demonstrated that patients with dominant TET2 mutations had significantly shorter OS than those with subclonal mutations, consistent with the initial findings (**Figure 5D**). These independent validation results further support the conclusion that dominant TET2 mutations are an adverse prognostic marker in AML and strengthen the external validity of our study.

## 4 Discussion

In this study, we performed a comprehensive analysis of 206 patients with cytogenetically normal acute myeloid leukemia to investigate the clonal characteristics of *TET2* mutations and their prognostic impact, with a particular focus on the clinical outcomes associated with dominant versus subclonal *TET2* mutations. Our findings not only reaffirm the significant association between *TET2* mutations and patient prognosis but also highlight the role of dominant *TET2* mutations as an independent adverse prognostic factor. These results provide novel insights into the molecular prognostic assessment of AML.

The detection frequency of *TET2* mutations in our cohort was 18.9%, which is consistent with previously reported mutation rates in AML ranging from 10–25% (13, 27, 28). Most *TET2* mutations were located between exons 3 and 11, with missense and frameshift mutations being predominant, aligning with prior studies (29). Patients with *TET2* mutations were significantly older than those without, corroborating earlier findings that *TET2* mutations are more common in elderly AML patients (20, 30). The higher prevalence of *TET2* mutations in older individuals may reflect that hematopoietic stem cell aging, chromosomal instability and reduced DNA repair efficiency accumulate with age (9, 10). Loss of *TET2* function has been shown to lead to age-related clonal expansion. As a DNA demethylation protein, *TET2* likely promotes AML cell proliferation by modulating the epigenetic landscape and oncogene expression (31). Partial or complete loss-of-function *TET2* mutations result in regional and contextual DNA hypermethylation, leading to gene silencing or activation, skewed myeloid differentiation, and clonal expansion. Thus, *TET2* mutations may not only serve as markers of clonal hematopoiesis of indeterminate potential but also contribute to leukemogenesis (32, 33).

Our cohort consists exclusively of rigorously defined *de novo* CN-AML with no documented antecedent myelodysplastic syndrome (MDS) or myeloproliferative neoplasm. Nevertheless, the extremely poor outcome of patients harboring concomitant TET2 and ASXL1 mutations (median OS only 9 months) strongly suggests the possible existence of an occult, clinically silent pre-leukemic phase in a subset of cases. TET2 and ASXL1 are canonical “founder” mutations in age-related clonal hematopoiesis and secondary AML (32, 33). It is therefore highly plausible that some TET2/ASXL1 co-mutated cases evolved from long-standing, undiagnosed low-blast-count MDS or clonal hematopoiesis of indeterminate potential (CHIP) that rapidly progressed to overt AML before the MDS phase became clinically detectable. This hypothesis is further supported by the observation that four of the six TET2/ASXL1 co-mutated patients exhibited excess marrow blasts approaching 20% and/or suggestive multilineage dysplasia in the months immediately preceding AML diagnosis. Thus, dominant clonal TET2 mutations—particularly when co-occurring with ASXL1—may delineate a biologically aggressive subgroup that shares key pathophysiologic features with secondary AML despite fulfilling strict diagnostic criteria for *de novo* disease.

Clonal evolution analysis revealed distinct patterns in AML (23, 24, 34). Dominant *TET2* mutations were associated with linear evolution, characterized by the acquisition of new mutations at relapse. In contrast, subclonal *TET2* mutations often exhibit branching evolution, with the disappearance of initial mutations upon relapse (24, 35). These findings suggest that dominant *TET2* mutations sustain clonal dominance through the continuous accumulation of new driver mutations, contributing to disease relapse and progression. Conversely, subclonal mutations may gradually lose their clonal advantage during disease progression, resulting in relatively better outcomes (26, 34–36). Our longitudinal analysis of clonal evolution, although limited to five patients, revealed distinct trajectories between dominant and subclonal *TET2* mutations. While the small sample size precludes definitive conclusions, the observed patterns—dominant *TET2* clones driving linear evolution and subclones displaying branching dynamics—align with prior single-cell studies in AML (23–26). This consistency suggests potential generalizability, but validation in larger cohorts with sequential sampling is critical to confirm these evolutionary models These differences in clonal evolution imply that patients with dominant *TET2* mutations may require more aggressive therapeutic interventions to prevent relapse. Importantly, the prognostic impact of *TET2* mutations is not determined by VAF but rather by *TET2* clonal dominance and the interplay between mutations within the same clone.

However, several limitations in our clonal evolution analysis should be acknowledged in this study. First, the resolution of clonal architecture was limited by the bulk sequencing approach used in this study (37, 38). Bulk sequencing cannot precisely delineate the heterogeneity of individual subclones or the temporal order of mutation acquisition when only a single detection is performed, which may lead to an incomplete understanding of clonal dynamics. Single-cell sequencing or other advanced techniques would provide a more detailed and accurate picture of clonal evolution (39–41). Second, the modest size of the TET2-mutated cohort (n=39), with further subdivision into dominant (n=27) and subclonal (n=12) cases, inevitably restricts statistical power for subgroup analyses of specific co-mutations. Although dominant TET2 clones exhibited a higher frequency of concomitant NPM1 mutations and TET2/NPM1 co-mutation was associated with inferior relapse-free survival, the limited number of cases precluded formal testing of whether the adverse outcome of TET2/NPM1 co-mutated disease is driven primarily by dominant TET2 clones. Similarly, the power to detect interactions between TET2 clonal status and other recurrent co-mutations (e.g., ASXL1, DNMT3A, NRAS) is low. Larger collaborative cohorts will be required to definitively resolve these potentially clinically important gene–gene interactions. Finally, the lack of longitudinal samples in some patients restricted our ability to fully track clonal evolution over time, these data are hypothesis-generating at best and do not permit any inference about distinct evolutionary trajectories of dominant versus subclonal TET2 mutations. Future studies with larger cohorts and single-cell resolution are needed to validate and extend our findings.

Survival analysis further demonstrated that dominant *TET2* mutations are an independent adverse prognostic factor for OS. This finding was independently validated through random sampling and analysis of the Beat AML database, which also showed significantly shorter OS in patients with dominant *TET2* mutations, reinforcing its potential clinical utility as a prognostic biomarker (13).

To facilitate individualized prognostication, we developed a nomogram model to predict the five-year survival probability of patients (42). Future studies are needed to validate this model across diverse AML subgroups and explore its utility in guiding treatment decisions.

## 5 Conclusion

This study systematically evaluated the clonal characteristics of *TET2* mutations and demonstrated that the prognostic impact of *TET2* mutations is not determined by VAF, but rather by *TET2* clonal dominance and the interplay between mutations within the same clone. These findings provide novel insights into the molecular pathogenesis of CN-AML.

## Data Availability

The raw data supporting the conclusions of this article will be made available by the authors, without undue reservation.
