# Supplementary material for: Dominant TET2 mutations predict adverse prognosis in cytogenetically normal acute myeloid leukemia patients
Source: Front Oncol. 2026 Jan 5;15:1730830. doi: 10.3389/fonc.2025.1730830 (PMC12812581; doi:10.3389/fonc.2025.1730830)
Supplement: Supplementary file 1 [file Table1.docx]

**Supplementary Table S1 Prediction of *TET2* Mutational Pathogenicity**

| Mutation Site | Mutaster Taster |  | AlphaMissense | |  | PolyPhen-2 | |  | Sorting Intolerant From Tolerant | |
| --- | --- | --- | --- | --- | --- | --- | --- | --- | --- | --- |
|  | Pathogenicity |  | Pathogenicity | score |  | Pathogenicity | score |  | Pathogenicity | score |
| **S1392R** | Disease causing |  | likely_pathogenic | 0.999 |  | Probably damaging | 1.000 |  | Affect protein function | 0.03 |
| **C1193W** | Disease causing |  | likely_pathogenic | 0.999 |  | Probably damaging | 1.000 |  | Affect protein function | 0.00 |
| **C1289F** | Disease causing |  | likely_pathogenic | 0.997 |  | Probably damaging | 1.000 |  | Affect protein function | 0.00 |
| **R1359C** | Disease causing |  | likely_pathogenic | 0.996 |  | Probably damaging | 1.000 |  | Tolerated | 0.13 |
| **R1214W** | Disease causing |  | likely_pathogenic | 0.979 |  | Probably damaging | 1.000 |  | Affect protein function | 0.00 |
| **L1886S** | Disease causing |  | likely_pathogenic | 0.978 |  | Probably damaging | 0.997 |  | Affect protein function | 0.02 |
| **E1178G** | Disease causing |  | likely_pathogenic | 0.978 |  | Probably damaging | 1.000 |  | Affect protein function | 0.00 |
| **N813S** | Polymorphism4 |  | likely_pathogenic | 0.074 |  | Benign | 0.006 |  | Affect protein function | 0.00 |
| **N1387S** | Disease causing |  | likely_pathogenic | 0.719 |  | Probably damaging | 1.000 |  | Affect protein function | 0.00 |
| **F775L** | Polymorphism |  | ambiguous | 0.462 |  | Benign | 0.000 |  | Tolerated | 0.75 |
| **P1655L** | Disease causing |  | likely_benign | 0.118 |  | Probably damaging | 0.983 |  | Affect protein function | 0.05 |

**Supplementary Table S2.**The clinical characteristic of different TET2 Variant Allele Frequency in CN-AML

| **Characteristics** | **TET2VAF≤43.36%**  **N=19** | **TET2VAF>43.36%**  **N=20** | ***P*** |
| --- | --- | --- | --- |
| **Age, *n* (%)** |  |  | **0.408** |
| ＜60 | 6 (31.5) | 10 (50.0) |  |
| ≥60 | 13 (68.5) | 10 (50.0) |  |
| **Sex, *n* (%)** |  |  | **0.242** |
| male | 12 (63.1) | 10 (50.0) |  |
| female | 7 (36.9) | 10 (50.0) |  |
| **Laboratory data ,**  **median (range)** |  |  |  |
| RBC (×10^12^ /L) | 2.58 (1.24-4.37) | 2.42 (1.17-3.86) | **0.638** |
| Hb (g/L) | 77 (46-136) | 79 (46-123) | **0.605** |
| WBC (×10^9^ /L) | 6.10 (0.89-115.20) | 23.01 (2.81-207.48) | **0.013** |
| PLT (×10^9^ /L) | 28 (6-269) | 43 (5-297) | **0.412** |
| BM blast (%) | 55 (21-93) | 54 (20-85) | **0.952** |
| LDH (U/mL) | 359 (95-1920) | 401 (203-2544) | **0.34** |
| **2022 ELN risk classification, *n* (%)** |  |  | **0.664** |
| Favorable | 2 (10.5) | 1 (5.0) |  |
| Intermediate | 14 (73.7) | 14 (70.0) |  |
| Adverse | 3 (15.8) | 5 (25.0) |  |
| **Outcome** |  |  |  |
| CR1**, *n* (%)** | 15 (78.9) | 11 (55.0) | **0.333** |
| RP1**, *n* (%)** | 9 (60.0) | 8 (72.7) | **0.680** |
| OS**,**  **median (range)** | 17 (2-36) | 24 (5-47) | **0.433** |
| RFS**,**  **median (range)** | 15 (6-25) | 20 (2-35) | **0.283** |

VAF:Variant Allele Frequency, RBC:Red blood cell, WBC:white blood cell, PLT:platelet, LDH: Lactate dehydrogenase, HBDH: -Hydroxybutyrate Dehydrogenase, ELN: European Leukemia Network, CR1: First Complete Remission，RP1:First Recurrence, OS:Overall Survival, RFS：Relapse-Free Survival

**Supplementary Table S3.** Univariate and multivariate Cox regression analysis for OS in AML patients.

| Characteristics | Total(N) | Univariate analysis | |  | Multivariate analysis | |
| --- | --- | --- | --- | --- | --- | --- |
|  |  | Hazard ratio (95% CI) | P value |  | Hazard ratio (95% CI) | P value |
| **Age** |  |  | 0.009 |  |  |  |
| <60 | 139 | Reference |  |  | Reference |  |
| ≥60 | 67 | 1.712 (1.135 - 2.581) | 0.010 |  | 1.499 (0.964-2.330) | 0.072 |
| **Gender** |  |  | **0.914** |  |  |  |
| Female | 106 | Reference |  |  |  |  |
| Male | 100 | 1.023 (0.681- 1.537) | **0.914** |  |  |  |
| **ELN2022** |  |  | **0.009** |  |  |  |
| Favorable | 22 | Reference |  |  | Reference |  |
| Intermediate | 151 | 2.455 (1.063 - 5.672) | **0.036** |  | 2.959 (1.153 - 7.586) | **0.024** |
| Adverse | 33 | 4.112 (1.624 - 10.411) | **0.003** |  | 9.091 (1.967-42.025) | **0.005** |
| **Allo-HSCT** |  |  | 0.096 |  |  |  |
| No | 198 | Reference |  |  | Reference |  |
| Yes | 8 | 0.300 (0.073 - 1.236) | 0.096 |  | 0.276 (0.066-1.149) | 0.077 |
| **Treatment** |  |  |  |  |  |  |
| “3+7” regimen | 145 | Reference |  |  |  |  |
| GAG | 61 | 1.290 (0.850-1.959) | 0.232 |  |  |  |
| **ASXL1**  **mutations** |  |  | 0.683 |  |  |  |
| WT | 186 | Reference |  |  | Reference |  |
| MUT | 20 | 1.155 (0.578 - 2.306) | 0.683 |  | 0.354 (0.082 - 1.523) | 0.163 |
| **CEBPA**  **mutation** |  |  | **0.138** |  |  |  |
| WT | 180 | Reference |  |  | Reference |  |
| MUT | 26 | 0.581 (0.281 - 1.200) | **0.004** |  | 1.187 (0.524 - 2.688) | 0.681 |
| **DNMT3A mutations** |  |  | 0.203 |  |  |  |
| WT | 163 | Reference |  |  |  |  |
| MUT | 43 | 1.341 (0.852 - 2.110) | 0.205 |  |  |  |
| **FLT3-ITD mutation** |  |  | **0.263** |  |  |  |
| WT | 170 | Reference |  |  | Reference |  |
| MUT | 36 | 1.341 (0.801 - 2.247) | **0.265** |  | 1.458 (0.798 - 2.663) | 0.220 |
| **IDH1 mutations** |  |  | 0.589 |  |  |  |
| WT | 195 | Reference |  |  |  |  |
| MUT | 11 | 1.256 (0.548 - 2.880) | 0.590 |  |  |  |
| **IDH2**  **mutations** |  |  | 0.795 |  |  |  |
| WT | 189 | Reference |  |  |  |  |
| MUT | 17 | 0.908 (0.439 - 1.880) | 0.795 |  |  |  |
| **NPM1 mutations** |  |  | 0.085 |  |  |  |
| WT | 151 | Reference |  |  | Reference |  |
| MUT | 55 | 0.690 (0.451 - 1.055) | 0.087 |  | 0.619 (0.388 -0.986) | **0.044** |
| **RUNX1 mutations** |  |  | 0.020 |  |  |  |
| WT | 203 | Reference |  |  | Reference |  |
| MUT | 3 | 3.609 (1.132 - 11.505) | 0.030 |  | 1.155 (0.188-6.318) | 0.923 |
| **TET2 mutations** |  |  | **0.016** |  |  |  |
| WT | 167 | Reference |  |  | Reference |  |
| Dominant | 27 | 2.130 (1.269 - 3.576) | **0.006** |  | 2.026 (1.036-3.964) | **0.039** |
| Subclone | 12 | 1.355 (0.521 - 2.281) | **0.818** |  | 1.027 (0.546 - 1.932) | 0.934 |
| **TP53**  **mutations** |  |  | **0.062** |  |  |  |
| WT | 201 | Reference |  |  | Reference |  |
| MUT | 5 | 2.856 (0.900 - 9.066) | **0.075** |  | 0.927 (0.166 - 5.170) | 0.931 |

HSCT, hematopoietic stem cell transplantation; ELN2017, 2017 European LeukemiaNet (ELN) classification guideline.

**Supplementary Table S4.** Univariate and multivariate Cox regression analysis for RFS in AML patients.

| Characteristics | Total(N) | Univariate analysis | |  | Multivariate analysis | |
| --- | --- | --- | --- | --- | --- | --- |
|  |  | Hazard ratio (95% CI) | P value |  | Hazard ratio (95% CI) | P value |
| **Age** |  |  | **0.004** |  |  |  |
| <60 | 139 | Reference |  |  | Reference |  |
| ≥60 | 67 | 1.899 (1.217 - 2.962) | **0.005** |  | 1.866 (1.118 - 3.115) | **0.017** |
| **Gender** |  |  | 0.858 |  |  |  |
| Female | 80 | Reference |  |  |  |  |
| Male | 77 | 1.023 (0.681- 1.537) | 0.858 |  |  |  |
| **ELN2022** |  |  | **0.013** |  |  |  |
| Favorable | 18 | Reference |  |  | Reference |  |
| Intermediate | 114 | 3.210 (1.287 - 8.009) | **0.012** |  | 3.410 (1.328 - 8.753) | **0.011** |
| Adverse | 25 | 4.165 (1.513 - 11.460) | **0.006** |  | 19.233 (4.149 - 89.160) | **<0.001** |
| **Allo-HSCT** |  |  | 0.289 |  |  |  |
| No | 198 | Reference |  |  | Reference |  |
| Yes | 8 | 0.538 (0.168 - 1.721) | 0.296 |  | 0.369 (0.109 - 1.256) | 0.154 |
| **Treatment** |  |  |  |  |  |  |
| “3+7” regimen | 145 | Reference |  |  |  |  |
| GAG | 61 | 1.433 (0.911-2.253) | 0.120 |  |  |  |
| **ASXL1 mutations** |  |  | 0.743 |  |  |  |
| WT | 140 | Reference |  |  | Reference |  |
| MUT | 17 | 1.118 (0.575 - 2.173) | 0.743 |  | 5.944 (1.470 - 24.036) | **0.012** |
| **CEBPA mutation** |  |  | **0.021** |  |  |  |
| WT | 138 | Reference |  |  | Reference |  |
| MUT | 19 | 0.598 (0.380 - 0.942) | **0.026** |  | 0.874 (0.507 - 1.505) | 0.626 |
| **DNMT3A mutations** |  |  | 0.467 |  |  |  |
| WT | 133 | Reference |  |  |  |  |
| MUT | 24 | 1.237 (0.696 - 2.198) | 0.468 |  |  |  |
| **FLT3-ITD mutation** |  |  | 0.130 |  |  |  |
| WT | 133 | Reference |  |  | Reference |  |
| MUT | 24 | 1.1.538 (0.873 - 2.696) | 0.133 |  | 1.295 (0.640 - 2.617) | 0.472 |
| **IDH1 mutations** |  |  | 0.226 |  |  |  |
| WT | 150 | Reference |  |  |  |  |
| MUT | 7 | 1.739 (0.702 - 4.306) | 0.231 |  |  |  |
| **IDH2**  **mutations** |  |  | 0.258 |  |  |  |
| WT | 144 | Reference |  |  |  |  |
| MUT | 13 | 0.598 (0.241 - 1.475) | 0.263 |  |  |  |
| **NPM1 mutations** |  |  | 0.095 |  |  |  |
| WT | 118 | Reference |  |  | Reference |  |
| MUT | 39 | 0.673 (0.442 - 1.075) | 0.097 |  | 0.578 (0.336 - 0.994) | **0.047** |
| **RUNX1 mutations** |  |  | 0.020 |  |  |  |
| WT | 156 | Reference |  |  | Reference |  |
| MUT | 1 | 1.466 (0.203 - 10.568) | 0.704 |  | 0.174 (0.015 - 1.982) | 0.159 |
| **TET2 mutations** |  |  | **0.021** |  |  |  |
| WT | 131 | Reference |  |  | Reference |  |
| Dominant | 18 | 4.165 (1.513 - 11.460) | **0.006** |  | 01.029 (0.524 - 2.021) | 0.934 |
| Subclone | 8 | 3.210 (1.287-8.009) | **0.012** |  | 1.120 (0.409 - 3.066) | 0.825 |
| **TP53 mutations** |  |  | 0.904 |  |  |  |
| WT | 154 | Reference |  |  | Reference |  |
| MUT | 3 | 0.886 (0.123 - 6.373) | 0.904 |  | 0.103 (0.09 - 1.233) | 0.075 |

HSCT, hematopoietic stem cell transplantation; ELN2017, 2017 European LeukemiaNet (ELN) classification guideline.
